# The Acute Effects of Motor Imagery Combined With Action Observation Breathing Exercise on Cardiorespiratory Responses, Brain Activity, and Cognition: A Randomized, Controlled Trial

**DOI:** 10.1155/cdr/6460951

**Published:** 2025-02-22

**Authors:** Ebrar Atak, Amine Ataç

**Affiliations:** ^1^Department of Physiotherapy and Rehabilitation, Faculty of Health Sciences, Yalova University, Yalova, Türkiye; ^2^Department of Physiotherapy and Rehabilitation, Faculty of Health Sciences, Istanbul Gedik University, Istanbul, Türkiye

**Keywords:** action observation, brain activity, cardiorespiratory responses, cognition, motor imagery

## Abstract

Breath and brain activity have been integral to daily life since time immemorial. Cognition and cardiorespiratory responses are closely interlinked, necessitating further investigation into their dynamics. The potential benefits of combining motor imagery (MI) and action observation (AO) based breathing exercises in rehabilitation have not been fully explored. This study was aimed at assessing the acute effects of MI combined with AO on cognitive function and cardiorespiratory responses. Thirty-three healthy adults were randomized into MI combined with AO breathing (MI+AO), active respiratory exercise (ARE), and control groups, with equal distribution across groups. Electroencephalography (EEG) data were collected using a Muse EEG headband, and cognitive function was assessed using the Montreal Cognitive Assessment (MoCA) while imagining activities were measured via the Kinesthetic and Visual Imagery Questionnaire (KVIQ). Significant improvements in the Timed Up and Go (TUG) test and systolic blood pressure were observed in the ARE group (*p* < 0.05), alongside improvements in MoCA and KVIQ scores (*p* < 0.05). EEG data revealed significant decreases in delta and theta power at the temporoparietal (TP) location in the ARE group (*p* < 0.05). These findings suggest that MI and AO, when combined with respiratory exercises, may serve as effective passive strategies to support cognition and cardiorespiratory function, particularly in individuals who struggle to actively participate in pulmonary rehabilitation.

**Trial Registration:** ClinicalTrials.gov identifier: NCT06099483

## 1. Introduction

Aging in humans is associated with a decline in cognitive and physical functions. While respiratory function has been suggested to predict long-term cognitive performance, emerging evidence indicates a bidirectional relationship. Cognitive decline is often reported through self-reporting of physical limitations. Specifically, changes in respiratory function—particularly in tasks requiring psychomotor speed and spatial abilities—can lead to declines in fluid cognitive function. Consequently, there is a significant relationship between decreased lung function and decreased cognitive function, emphasizing the importance of maintaining respiratory function to ensure cognitive performance [1]. Studies have shown that impairments in respiratory function are generally associated with poorer cognitive performance across all cognitive measurements. This suggests that lung function could be a relevant factor to consider in experimental studies related to cognitive functions in healthy adults [2]. Furthermore, lower respiratory capacity has been linked to cognitive impairments and an elevated risk of depression. This implies that individuals with higher respiratory capacity have better cognitive functions, which in turn reduces the risk of depression [3]. Similarly, in conditions such as chronic obstructive pulmonary disease (COPD), which impairs pulmonary functions, improving exercise capacity has been shown to enhance cognitive function and delay cognitive decline [4]. Cognitive states are also affected in cardiovascular diseases or healthy individuals at risk [5, 6]. As evidenced in the literature, the greater the impact on pulmonary functions, the higher the likelihood of severe cognitive impairment.

The concept of action simulation refers to the internal representation of motor programs without the execution of explicit movements. Motor imagery (MI) and action observation (AO) are recognized as two distinct methods of action simulation that activate motor areas in the brain. According to Jeannerod's action simulation theory (AST), individuals mentally simulate actions without physically performing them. Jeannerod proposed that when people imagine or observe an action, their brain activates similar neural pathways as if they were actually performing the action. This mental rehearsal, or “MI,” involves creating a covert representation of the action, including its goals, means, and consequences. These simulations occur both when people plan their actions and when observing others perform them. Such methods are critical for understanding and facilitating motor processes without physical movement, as they engage neural pathways typically used during motor execution [7, 8]. AO and MI therapies have become increasingly popular as new rehabilitation methods in recent years, particularly pertaining to stroke, Parkinson's, mental and cognitive rehabilitation, spinal cord injury, and orthopedic rehabilitation [9, 10]. AO involves real-time motor simulation, where observing another person's behavior triggers neural activity associated with that movement [11]. MI involves participants imagining themselves performing a movement as instructed, without actually engaging any muscle pathways. It is a mental simulation of physical action [10]. The rationale behind these rehabilitative interventions is the activation of the specific mirror neuron system in the premotor, motor, and parietal cortices of the brain while observing and imagining actions [11]. By simply performing MI, a similar reaction to the active area of the brain activated during physical training can be achieved, which can provide similar benefits to physical training in all age groups.

In addition, the biggest advantage of MI is its cost-effectiveness. It enhances performance by stimulating motor networks, increasing sensory-motor integration, reducing cognitive load, and strengthening the mind–body connection [12]. Through limited literature and the use of imaging techniques like electroencephalography (EEG), functional magnetic resonance imaging, positron emission tomography, and transcranial magnetic stimulation, both invasive and noninvasive, brain regions with mirror activation properties have been discovered in humans during the activation of these methods [9].

Motor imagery and action observation (MIAO) methods can influence respiratory rate, heart rate, and skin temperature due to sympathetic nervous system activation. Increases in electrodermal activity have also been reported. Evidence suggests that during mental practice, brain regions controlling the autonomic nervous system are activated [8, 13]. Unlike traditional MI or AO alone, AO+MI simultaneously engages both visual and motor systems. As noted by Cuenca-Martínez et al., this integration enhances sensorimotor engagement and optimizes motor network activation compared to using MI or AO in isolation [14]. The sympathetic pathways to the heart are modulated by the activity of the anterior cingulate cortex, and the cardiovagal activity is under the control of the ventral medial prefrontal cortex. Some studies have reported respiratory and hemodynamic changes in response to mental simulation exercises. Comprehensive studies have emerged in recent years, examining cardiorespiratory responses during MI as part of health and performance strategies [15–19].

EEG is a noninvasive method used to distinguish a variety of human behaviors by monitoring neurological responses during cognitive and motor tasks [20]. As a learning tool, MI facilitates neural network engagement, with significant implications for rehabilitation and empirical research [21].

Analyzing brain neural activity is a seemingly indispensable tool for quantitatively evaluating interactions among neurobiological systems. Brain networks synchronize unilaterally or bilaterally during cognitive, motor, and action simulation tasks [22]. However, the high cost of traditional laboratory EEG systems has limited their accessibility to researchers. This challenge has spurred interest in expanding EEG portability, even for home-based settings. Brain–computer interfaces (BCIs) now allow direct communication between the brain and external devices, and portable EEG systems have been validated for use in brain activity imaging studies [23–25].

In the literature, there have been a limited number of studies using AO and MI methods in the treatment and rehabilitation of various pathologies, focusing on their chronic effects on brain activity [26, 27]. As far as can be ascertained, there has to be a randomized controlled study that investigates the acute effects on brain activity, cardiorespiratory responses, and cognitive function of AO and MI methods when applied in the form of respiratory exercises in healthy individuals. This study fills that gap.

The aim of this study is to investigate the acute effects of respiratory exercises applied as a combined AO and MI intervention. By doing so, the study seeks to evaluate their impact on cognitive functions and cardiorespiratory responses in healthy sedentary individuals. The ultimate goal is to develop alternative methods that could address the interconnected issues of cognitive decline and cardiorespiratory problems, based on the results obtained.

## 2. Materials and Methods

### 2.1. Study Design

This study was a prospective, single-center, randomized controlled trial with concealed allocation. It was carried out under the supervision of the local ethics committee (protocol number: E-56365223-050.02.04-2023.137548.176-550). The protocol adhered to the principles of the Declaration of Helsinki [28]. A written informed consent was obtained from each participant.

### 2.2. Participants and Inclusion Criteria

A total of 33 healthy adults participated in this study. Participants were randomly assigned to one of three groups: the MI combined with AO group (MI+AO), the active respiratory exercise (ARE) group, or the control group (CGr), using a 1:1:1 ratio generated by an online randomization tool (https://www.randomizer.org).

Participants aged 18–45 years were eligible if they

• voluntarily agreed to participate;
• did not use any assistive devices;
• had no comorbidities affecting the orthopedic, neurological, or cardiac systems;
• scored 30 or more on both visual and kinesthetic imagery sections of the Kinesthetic and Visual Imagery Questionnaire-20 (KVIQ-20); and
• scored 26 or higher on the Montreal Cognitive Assessment (MoCA) test.

Exclusion criteria included individuals who

• declined participation,
• used oral corticosteroids [29, 30] within the last 4 weeks,
• were unable to understand verbal instructions or had visual impairments, and
• participated in other clinical studies within the past 30 days that could affect the outcomes.

Participants were examined by a physician to ensure they met the inclusion criteria. Corticosteroid use was allowed only if it occurred at least 4 weeks prior to the study and was unrelated to organ transplantation.

## 3. Procedures

The environment for the evaluations was carefully controlled to be free of external distractions. EEG recordings were conducted in a noise-free room, free from electrical devices, to ensure data accuracy. Participants were asked to remain as still as possible during measurements. The device only has electrodes corresponding to the anterior frontal (AF) 7 and 8 and temporoparietal (TP) 9–10 areas and receives EEG signals from these channels. Frontal and temporal areas are areas responsible for sensory and motor processes.

While the device's limited electrode count may not capture pathological processes in detail, it provides sufficient data to evaluate brain activity patterns and functional gradations. Future studies will incorporate devices with at least 16 channels for more comprehensive data. However, Muse 2 was selected for this study due to its ease of use, affordability, and portability.

Each group session lasted 10 min, and assessments of brain activity, cardiorespiratory responses, and cognitive functions were performed both before and after the session. The applications were done in a quiet room. No prior preparation was made to prevent any learning effects. A pre-prepared video recording was viewed by each participant on a laptop screen for 10 min. Each participant sat upright in an office chair with hands placed on their laps. The laptop was placed on a square table (1 m) with a red-colored surface. EEG measurements were recorded while imagining was being done.

### 3.1. MIAOGr

**Figure 1:**
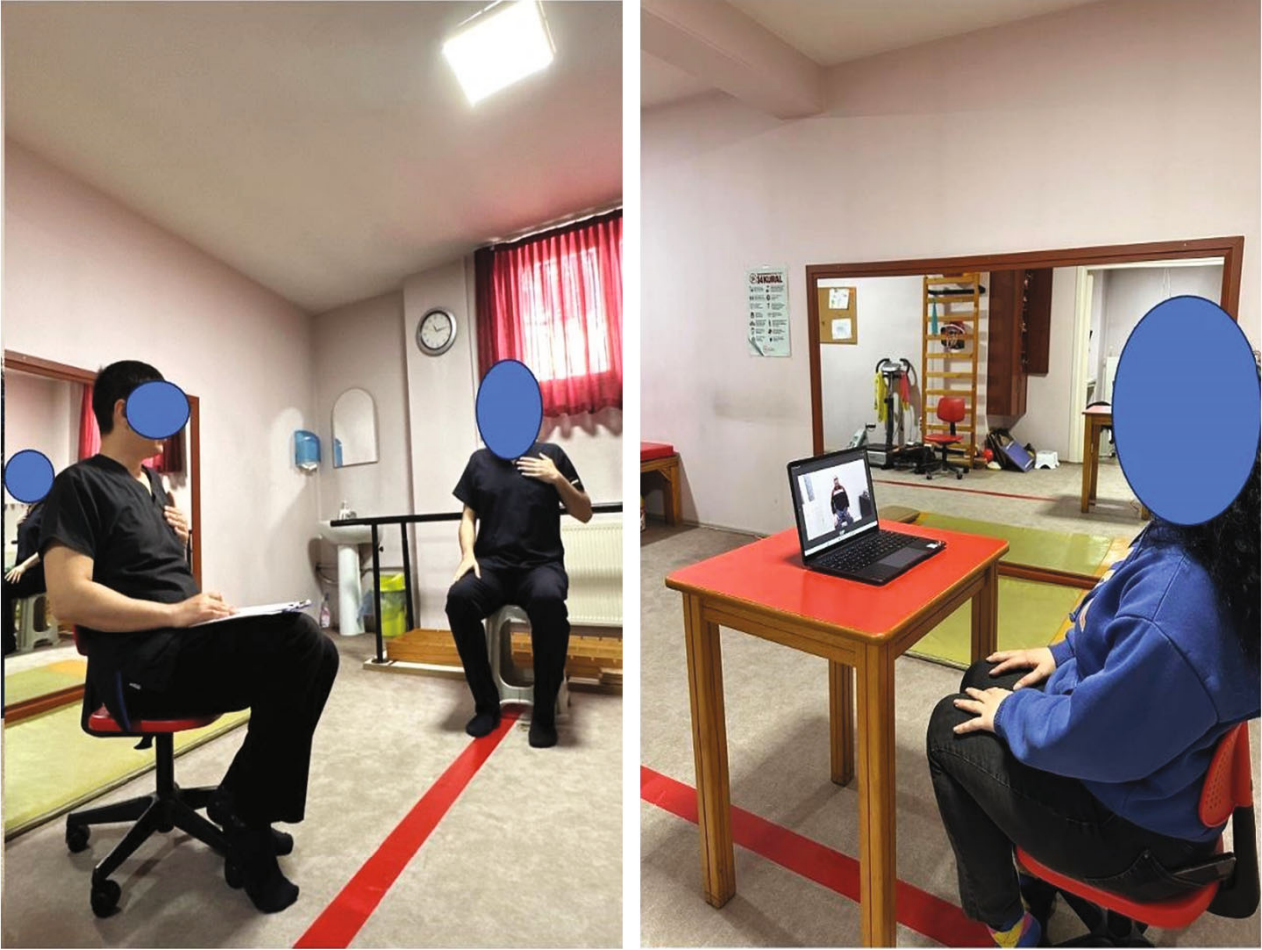
Illustration of procedures.

Participants in the study were subjected to breathing control, diaphragmatic breathing, and thoracic expansion exercises using the MI+AO methods [31]. These exercises were presented in sets, each with a corresponding number of repetitions (10 repetitions each), captured on video from front-lateral angles. The participants were instructed to watch the videos, maintain a distance of 60 cm from the screen, follow the instructions, and mentally visualize themselves performing the exercises as demonstrated. [32]. Participants were instructed to imagine each exercise as if they were performing them, following the given commands to enhance the feeling of reality. During AO, each exercise was played for 90 s in the video, with 30-s breaks between each exercise. In MI, participants were asked to imagine each exercise for 45 s as if they were doing it, followed by a 15-s rest, with appropriate commands given for each phase [33]. During the sessions with commanded videos where no active body movement occurred, EEG measurements were taken using the Muse 2 headset. This allowed for the recording of brain neurophysiological activity over the duration of the 10-min session (Figure 1).

### 3.2. AREGr

In this group, participants were taught and actively performed breathing control, diaphragmatic breathing, and thoracic expansion exercises with eyes open in a resting state prior to the session. Each exercise was performed for 10 repetitions. Since active body movements occurred in this group, Muse 2 headphones were used in EEG measurements in order not to restrict the movement capabilities of the participants. In order not to affect the data quality of active movements, the exercises were taught to the person before the session and were shown to the person by the practitioner with silent, command-free body mimics throughout the session. The occurrence of active movements can be considered a limitation of the study. The brain's neurophysiological activity was recorded throughout the 10-min session [23] (Figure 1).

### 3.3. CGr

Like the other groups, individuals in this group were seated with their eyes open for 10 min without any intervention. During this time, spontaneous EEG recordings were taken.

All interventions and measurements were conducted daily between 5:00 PM and 6:00 PM over 1 month to maintain consistency. The objective of following identical time slots was to ensure consistency. They were conducted between 5:00 PM and 6:00 PM within the span of 1 month. The objective of following identical time slots was to ensure consistency.

## 4. Outcomes

### 4.1. Brain Wave Activity Measurement

#### 4.1.1. EEG Measurement

The Muse 2 headband was utilized for its nonrestrictive design and ease of setup [23]. EEG measurements were collected before and during the MI+AO sessions. Kinesthetic and Visual Imagery Questionnaire (KVIQ) measurements were collected before and after the MI+AO sessions. In all three groups, 10-min EEG recordings were taken for participants during MIAO, active exercises, and at rest in the CGr, using the Muse 2 headband (Muse Version: 2016, InterAxon Inc., Toronto, ON, Canada). The EEG device operated at a sampling rate of 256 Hz, with the Fpz electrode serving as the reference. Four active electrodes—TP9, AF7, AF8, and TP10—were placed in their respective positions [34].

Prior to each EEG measurement, the equipment was carefully cleaned with an industry-compliant solution, and subsequently, the EEG sensor was placed on the participants' foreheads and ears. An Android tablet with the MindMonitor application was used. The software displayed acceptable impedance values for each electrode as green indicators on the interface, signalling readiness for recording. Raw EEG data were collected under these conditions. If the signal was found to be noisy upon visual inspection, the headband's position was adjusted.

The variance per second for each EEG channel was examined to determine signal quality, and data collection began when the variance per second for all channels was less than 200. Finally, our specialized software calculated the number of trials lost per experimental block in real time. If a block had more than 50% lost trials, Muse 2 was adjusted to improve signal quality, and then each block was repeated. The raw data were windowed using a Hanning filter. The Muse Monitor software automatically removed artifacts during recording. Each data file underwent additional processing with a fifth-order Butterworth filter, applying a band-pass filter in the 0.5–40 Hz range. Finally, the data were analysed using a fast Fourier transform (FFT) procedure [35].

### 4.2. Measurement of Cardiorespiratory Responses

The assessment of cardiorespiratory parameters included the following measurements: respiratory frequency, blood pressure measured using a digital OMRON (Hoofdrop, Netherlands) blood pressure monitor, heart rate, and oxygen saturation level (SpO2) measured using a pulse oximeter.

### 4.3. Measuring Cognitive Abilities

#### 4.3.1. KVIQ

The KVIQ is a valid and reliable tool used to assess imagery ability in a seated position. It consists of a total of 20 items, including 10 visual and 10 kinesthetic items and min/max scores. Cronbach's alpha value of the questionnaire is satisfactory (test–retest: 0.98–0.98). This value was found to be 0.95 for the test and 0.95 for the retest for visual items and 0.97 for the test and 0.96 for the retest for kinesthetic items. Intraclass correlation coefficients for visual items ranged between 0.61 and 0.90 with a 95% confidence interval (CI) and between 0.59 and 0.89 for kinesthetic items. Factorial analyses showed that two factors explained 64.21% of the total variance [26, 27]. Participants were instructed according to the questionnaire, and they were explicitly informed that there should be no active movement during imagery. They were asked to indicate their imagery levels on the scale provided [36–38].

#### 4.3.2. Mental Chronometer Evaluation With Timed Up and Go (TUG) Test

The temporal synchronization of real and imagined movements was assessed using the TUG test to evaluate the temporal alignment between real and imagined movements [37]. The protocol for the TUG test was simulated for imagery ability and measured using the mental chronometry method. The temporal alignment between the actual and imagined TUG was calculated in terms of delta time using the formula “[(TUG actual time − TUG imagined time)/[(TUG actual time + TUG actual time/2)]] × 100” [28, 38]. The delta time calculated with the mental chronometry is indicative of the function of higher centers associated with walking controlled by cognitive processes. A higher delta time suggests lower MI ability [39, 40].

#### 4.3.3. The MoCA

The MoCA consists of various items that assess visual construction, executive function, memory, language, orientation, and attention areas. It is easily administered and can be scored with a maximum of 30 points [41]. This test assesses visual-spatial (5 points), naming (3 points), attention (6 points), language (3 points), abstraction (2 points), memory (5 points), and orientation (6 points) abilities. Possible scores range from 0 to 30 points, with higher scores indicating better cognitive function [42]. A score below 26 indicates cognitive impairment [43]. Permission to use the MoCA has been obtained for this study.

### 4.4. Statistical Analysis

SPSS software computer programme (Version 25, United States) and statistical software were used for the statistical analyses. Demographic data and comparisons between groups for smoking status were conducted using the Kruskal–Wallis test and the chi-square test. For each group, mean, standard deviation (SD), and minimum-maximum values were calculated. Pre- and postapplication clinical measurements and tests for each group were analysed using the Wilcoxon test. EEG analyses were conducted using repeated measures ANOVA, considering Greenhouse–Geisser values. In the ANOVA design, groups (MI-AO, ARE, and CGr) were defined as between-subjects factors, application time (two: preapplication and during application), frequency (five: delta, theta, alpha, beta, and gamma), location (two: AF and TP), and hemisphere (two: left and right) were defined as within-subjects factors. Post hoc analyses were performed using the Bonferroni test. The significance level was accepted as *p* < 0.05 in all tests.

#### 4.4.1. Power Analysis

The number of patients who should be included in the sample in the study was carried out using the data from the study conducted by Nicholson et al. [44]. The similarity of demographic and physical characteristics comprised education disparity, gender association, and smoking habits. As a result of the calculations, we estimated that a total of 27 participants were needed to achieve 95% power with a 5% Type 1 error.

## 5. Results and Discussion

### 5.1. Demographic Characteristics

When looking at the average ages for the groups, MI-AO had an average age of 42.42 (SD: 12.87), ARE had an average age of 24.10 (SD: 10.99), and CGr had an average age of 31.91 (SD: 10.83). The average height values for the groups were ARE 1.66 (SD: 0.09), ARE 1.72 (SD: 0.10), and CGr 1.67 (SD: 0.07). The average weight values were MI-AO 71.33 (SD: 6.98), ARE 70.50 (SD: 20.59), and CGr 63.54 (SD: 9.75). When looking at the average number of cigarettes smoked per day for the groups, ARE had an average of 6.75 (SD: 9.52), ARE had an average of 1.10 (SD: 3.48), and CGr had an average of 3.27 (SD: 5.08). Regarding the number of years of smoking, MI-AO had an average of 7.42 years (SD: 10.85), ARE had an average of 0.50 years (SD: 1.58), and CGr had an average of 3.64 years (SD: 8.89). In terms of gender distribution, MI-AO had eight females (four males), ARE had three females (seven males), and CGr had seven females (four males).

### 5.2. Differences in Measurements Between Groups and Acute Response

The Kruskal–Wallis test analysis did not show any statistically significant differences between the groups in the initial measurements (*p* > 0.05). However, in the final measurements, it was observed that there was a significant difference only in the physical time of the TUG test (*p*: 0.036) (mean ± SD (min–max) MI-AO: 7.01 ± 1.23 (5.80–10.39); ARE: 6.18 ± 0.81 (5.21–8.20); CGr: 7.17 ± 1.29 (6.02–10.41)). The first and last measurement results obtained with the Wilcoxon test are shown in Table 1.

**Figure 2:**
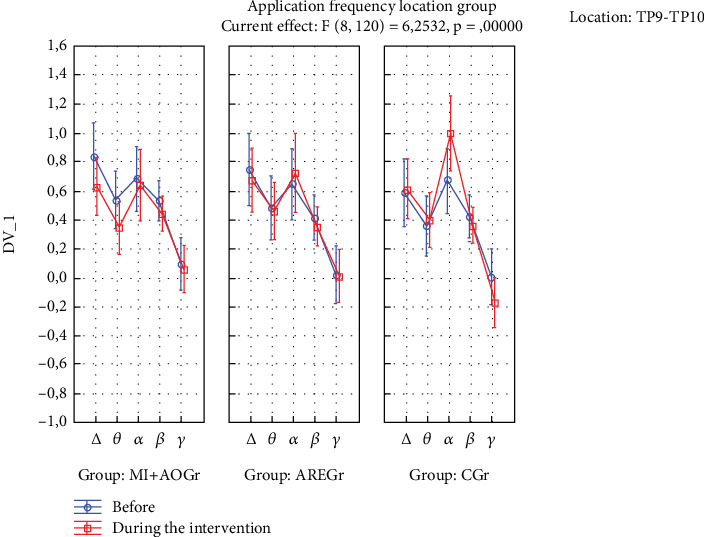
EEG results for TP9 and TP10 regions.

**Figure 3:**
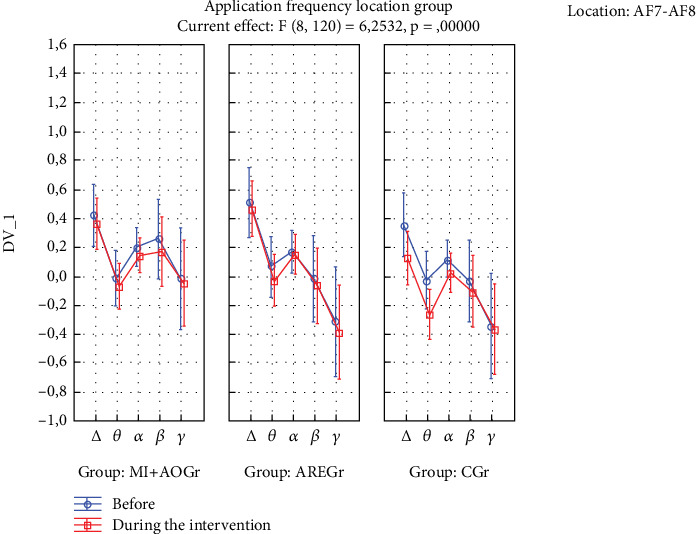
EEG results for AF7 and AF8 regions.

The time of application × frequency × location × group interaction was statistically significant (*p* < 0.05). In the ARE group, there was a decrease in delta and theta power in the TP location during the application compared to before (*p* < 0.05), while this was not observed in the other groups (*p* > 0.05) (Figure 2). In the CGr, there was a decrease in delta and theta power in the AF location during the application compared to before (*p* < 0.05), while this was not observed in the other groups (*p* > 0.05). Additionally, in the CGr, there was an increase in alpha power in the TP location during the application compared to before (*p* < 0.001), while this increase was not observed in the other groups (*p* > 0.05) (Figure 3).

## 6. Discussion

This study compared the brain waves, cognitive abilities, and cardiorespiratory responses of healthy individuals during MI with AO, AREs, and while doing no activity. Most studies do not focus on the intersections of cardiorespiratory responses and cognitive changes. Additionally, many studies primarily analyze changes in muscle activity using electromyography (EMG) rather than using EEG for brain analysis. Even when EEG analysis is conducted, it often does not focus on comparing exercise therapies but instead focuses on real-time movement observations for interface development [45–50]. One notable difference in our study compared to previous research on MIAO is the incorporation of AREs without active movement in the design of MIAO methods. This study implemented and actively administered respiratory exercises, which, to the best of our knowledge, had not previously been performed. Additionally, a CGr was included in the study to compare the effects of these methods on cardiorespiratory responses, cognitive abilities, and brain waves.

According to our results, the MI+AO method produced a statistically significant acute improvement in blood pressure among cardiorespiratory responses. Both MI+AO and actively administered respiratory exercises yielded statistical improvements in cognitive abilities. In terms of EEG results, the MI+AO group exhibited significantly lower delta and theta power at the TP location during the application compared to before. This suggests a neurocognitive advantage for the MI+AO group over the other groups because an increase in delta and theta waves is commonly observed in neurocognitive disorders, and theta rhythm is associated with emotional stress and is present in sleepy adults [51–55]. The observation of an increase in alpha power at the TP location in the CGr compared to before the application, while not observed in the other groups, supports the suggestion of comparing our study with more advanced EEG measurement systems in further research.

The relationship between cardiorespiratory responses and pulmonary functions is known. Improvement in pulmonary functions, especially, positively affects right heart functions and cardiorespiratory responses [56, 57]. Improvement in pulmonary functions not only enhances cardiorespiratory responses but also contributes positively to cognitive processes. The findings obtained in our study support this. In their study published in 2021, Grosprêtre et al. conducted comprehensive research on the physiological responses during MI, highlighting that no previous study had thoroughly explored these responses. They specifically investigated the acute effects of the MI method during sitting and standing on the autonomic nervous system and cardiometabolic responses [15]. They found that performing MI during standing resulted in greater spinal excitability and sympathetic activation in EMG results compared to sitting. This led to increased oxygen consumption, energy expenditure, ventilation, and lower cardiac output during standing MI. In our study, we investigated the acute effects of MI+AO during a seated respiratory exercise session without active movement. We examined its impact on cardiorespiratory parameters, cognitive functions, and brain waves, comparing it to an active exercise group and a CGr. Similar to the findings in the study by Grosprêtre et al., we observed a decrease in blood pressure during MI in both seated and standing positions. This suggests that MI can have a beneficial effect on blood pressure regulation regardless of body posture. When assessing spinal flexibility through muscle activities in both standing and sitting postures, they stated that the MI method made a positive contribution. In our study, the TUG test was performed to introduce a variation for use in the mental chronometer method, and statistically, it was observed that physical performance increased in the ARE and MI+AO groups. This could be attributed to the fact that, as expressed in the literature, MIAO methods are promising approaches to maintaining or increasing muscle strength, contributing to motor skill acquisition, and leading to enhanced physical performance [49]. No significant differences were observed among the groups in other cardiorespiratory parameters. In our study, the decrease in blood pressure parameters only in the ARE group may indicate a positive effect of the method on blood pressure. The absence of significant changes in other cardiorespiratory parameters could be attributed to processes related to goal selection, high-level planning, plan encoding, and plan execution, as they are not influenced by the method [57, 58].

Higher initial lung function is associated with a lower rate of cognitive decline in older adults independently. Studies have shown that the positive relationship between respiratory function and cognition is stronger in women, individuals with lower levels of education, or those who have never smoked [58]. Looking at the results of our study, it is understood that the outcomes obtained from both the active and the MI+AO respiratory exercise groups have provided positive contributions to respiratory functions and cognitive functions. The findings of a study examining the relationship between respiratory functions and cognitive abilities in young and middle-aged individuals highlight the importance of respiratory volumes for cognitive functions in later years. In this study conducted by Vasilopoulos et al., it was stated that early adult cognitive ability could be an indicator of many parameters of respiratory function related to aging 35 years later, including lung volume, airflow, and ventilatory capacity [59]. It was mentioned that cognitive impairments associated with the deterioration of lung functions due to aging could, therefore, be partly pre-existing. However, the results also emphasize that early-life risk factors may be differently associated with measurements of lung health in later life. Our study's findings have shown that active or passive interventions aimed at improving respiratory functions in individuals aged 18–45 years, whether they smoke or not, have been effective in suppressing EEG theta and delta frequencies. This effect is even more pronounced in the MI+AO group. The positive changes in EEG findings, along with improvements in KVIQ, MoCA, and mental chronometer (TUG) results, suggest that respiratory exercises also regulate electrical activity in the brain. Ultimately, active or passive respiratory exercises in healthy adults offer promising results in enhancing both respiratory capacity and cognitive functions [60]. It is indicated that weak lung functions are associated with mild cognitive impairment. Individuals with low lung function are reported to be in need of early cognitive evaluation. Furthermore, it has been shown that in lung diseases where respiratory functions are affected, improving cognitive status through cognitive rehabilitation methods has positive effects on quality of life, functional abilities, and respiratory capacities [61]. In the literature, there are studies aiming to show the degree of effectiveness of environmental and genetic factors on respiratory functions. In this context, one study has indicated that the FEV1 (forced expiratory volume in 1 s) value is an indicator of fluid intelligence [62]. Within the scope of our study, the respiratory exercises applied have positively changed cardiorespiratory responses, and it has been determined that this also provides a positive contribution to cognitive functions. The results of our study, which investigated the effects of pulmonary rehabilitation techniques on cardiorespiratory and cognitive functions, support the literature indicating that respiratory exercises performed actively or through MI+AO have positive effects on cognitive functions.

When we examine the EEG results in our study, we can see different outcomes. Particularly, the suppression of delta and theta rhythms at the TP location is a noteworthy finding. As is known, lower levels of delta and theta waves in adult individuals are indicative of better neurocognitive processing [50–54]. In our study, significant decreases in delta and theta waves were observed only in the TP region for the ARE group, while a significant decrease was observed only in the AF region for the CGr. This difference may be attributed to the possibility that the interventions in the ARE groups may have suppressed the AF region and, therefore, may not have led to a significant decrease in delta and theta waves. Since no intervention was applied in the CGr, this region may not have been suppressed. From this perspective, it can be considered that AREs may have the potential to suppress both TP and AF regions, possibly leading to a significant decrease in these waves. Additionally, the increase in alpha power in the TP region before and after the intervention in the CGr can be interpreted as the interventions in the ARE groups suppressing the alpha rhythm. Studies have shown that the suppression of the alpha rhythm is associated with positive changes in attention-related cognitive processes. The absence of an increase in alpha power in the other groups may be due to the possibility that, during MI+AO and AREs, mental plan encoding on one side could have suppressed it. This suggests that the mental processes involved in plan encoding during these activities may have led to the suppression of alpha rhythms in these groups [52–64].

The results of the study conducted by Çiftçi and Yılmaz in 2024 align with previous research suggesting that AO and MI may influence cognitive and perceptual outcomes, even though physical performance improvements are not always consistent. AO and MI interventions did not produce significant changes in jump performance but improved participants' perception of their abilities, highlighting the importance of cognitive engagement and self-efficacy in performance enhancement. Similarly, other studies have emphasized the role of physical experience and video observation in motor learning and technique development. Visual inputs have been proposed to support the formation of effective motor representations. The findings underscore the importance of designing tailored imagery protocols that consider individual prior experiences and the nature of motor tasks [65]. In our study, the suppression of delta and theta waves in the MI+AO group and the increase in alpha waves in the CGr suggest that the conducted interventions may have a positive impact on cognitive processes, particularly those related to attention. Our findings align with the results of the study conducted by Çiftçi and Yılmaz.

The nonsuppression of delta and theta in the TP location for the ARE group could potentially be advantageous in the rehabilitation of both healthy individuals and those with certain conditions or pathologies. When looking at the overall picture, it may be preferable to use ARE in situations where performing respiratory exercises is difficult for various reasons. Sugino and Ushiyama's study examined differences in EEG sensorimotor rhythms between gymnasts and non-gymnasts during imagery of non-sport–specific movements only in upper extremity movements (i.e., wrist dorsiflexion and shoulder abduction) [31]. The current physiological findings may be due to differences between gymnasts and non-gymnasts in the ability to flexibly modulate corticospinal excitability when imagining their own movements in reference to actual movement, they said. In our study, EEG recordings during different applications were examined only in healthy participants. When we look at the literature, it is seen that the MI method has been studied mostly in neurological diseases and athletes. In particular, variations in participant characteristics, applied methods, and measurement techniques may contribute to inconsistencies in EEG results reported in the literature. In this context, more studies are needed on AO and MI methods. Furthermore, conducting similar studies with more advanced EEG systems on both healthy individuals and those with cognitive impairments is important for understanding the effects of active exercise, MI, and AO applications.

In consideration of the tools available during further study, the BCIs allow direct communication between the brain and a microchip-controlled device. As BCI technology becomes more popular, mobile and affordable EEG devices have been validated in the literature for their utility and potential and thus are starting to be used in brain activity imaging studies [25, 45].

One of the important limitations of this study is that the number of channels of the EEG device used to make breathing exercises comfortable is low. Although it has the necessary software infrastructure to eliminate frequency ranges and noise pollution, it is important to conduct similar studies using EEG devices with more channels in order to determine the effectiveness of the applications with fashioned objectivity and added rigor. These limitations that arise inherent to the Muse device are because there are fewer channels to capture spatial and regional brain activity, which in turn can naturally impact the quality, granularity, and interpretation of the data. More specifically, the next stage of the study will prioritize precluding the following potentially detrimental aspects of the EEG measuring process—missing signals from key areas, reduced spatial resolution, low signal-to-noise ratio, and multiregional analysis (e.g., cortical regions). Indeed, these shortcomings in the procedure can influence and result in misinterpretation of data as well as utility in clinical applications.

The need for more advanced EEG systems in future studies is undeniable. We strongly recommend and emphasize that researchers use more advanced EEG systems with a greater number of channels to avoid the limitations posed by systems with restricted channel capacity. This improvement would allow for more comprehensive data collection and analysis.

Additionally, while the age range of 18–45 is considered young according to the World Health Organization (WHO) data, the wide age range in this study can be considered a limitation. In our study, we examined the effects of our methods on cardiorespiratory and cognitive parameters, but it is important to note that as age increases, risk factors in cardiovascular health may increase, and cardiovascular and cognitive health may interact and influence each other [66].

Since the cardiorespiratory health of a person in their 20s may not be comparable to the cardiorespiratory health of a person in their 40s, we recommend that future studies consider using narrower age ranges or conducting comparisons between distinct age groups. Another limitation of this study is its small sample size, which affects the generalizability and statistical power of the findings. Although power analyses were performed, we recommend that future studies be conducted with larger sample sizes to enhance reliability and validity.

## 7. Conclusions

This study primarily focused on acute interventions; however, it provides significant implications for future research. Breathing exercises applied through MIAO methods without physical movement have been shown to positively contribute to blood pressure, physical adaptation, and cognitive abilities. In addition to the methods utilized in this study, it is hoped that similar research exploring the effects of specific cognitive disorders and their potential impacts on cardiorespiratory problems will emerge.

Within this context, passive methods such as MIAO in respiratory rehabilitation have been observed, at least in a study group, to be effective and efficient in enhancing cardiorespiratory capacity and cognitive functions. Consequently, this study highlights the potential of MIAO as innovative, passive, and noninvasive strategies for respiratory rehabilitation and cognitive development.

The notable improvements observed within a controlled group of individuals suggest a paradigm shift in therapeutic applications, offering a new pathway for effectively and efficiently strengthening cardiorespiratory capacity and cognitive functions. As the field progresses, MIAO methods could become integral components of personalized rehabilitation programs, enabling patients and clinicians to achieve better health outcomes.

MIAO methods can serve as alternative rehabilitation strategies for individuals who cannot tolerate active exercise programs or who exhibit low motivation to engage in active exercise at the initial stages of rehabilitation. In our study, these methods demonstrated potential as viable alternatives to achieve cardiovascular and physical benefits, as they activate neurophysiological and neuroanatomical regions associated with active movement through observation and imagery, without requiring actual physical movement.

Future studies could enrich the literature by comparing the effects of these methods across larger sample sizes, diverse disease groups, and varying age ranges. We recommend incorporating MIAO methods alongside active exercises, particularly for individuals who struggle to tolerate or initiate active exercise programs. These methods may be especially valuable for triggering cardiovascular and cognitive benefits or preventing declines in physical and cognitive capacity.

## Figures and Tables

**Table 1:** Differences in measurements between groups and acute response.

**MI-AO (** **N** = 12**)**				**ARE (** **N** = 10**)**			**CGr (** **N** = 11**)**		
	**Before (** **m** **e** **a** **n** + **SD) (min–max)**	**After (** **m** **e** **a** **n** + **SD) (min–max)**	**p**	**Before (** **m** **e** **a** **n** + **SD) (min–max)**	**After (** **m** **e** **a** **n** + **SD) (min–max)**	**p**	**Before (** **m** **e** **a** **n** + **SD) (min–max)**	**After (** **m** **e** **a** **n** + **SD) (min–max)**	**p**
SBP	120.1 ± 11.3 (102–143)	115 ± 9.5 (106–138)	**0.04**	118.7 ± 16.8 (100–159)	102.5 ± 35 (11–135)	0.386	109.6 ± 12.5 (86–130)	104.7 ± 12.2 (81–117)	0.155
DBP	86 ± 31.7 (64–184)	76.4 ± 7.5 (55–83)	0.4	76.2 ± 9.6 (65–97)	72.5 ± 13.5 (51–90)	0.308	67.8 ± 12.7 (46–88)	66.8 ± 12.1 (44–83)	0.563
Pulse	85.4 ± 14.3 (58–108)	80.6 ± 7.8 (66–93)	0.07	83.8 ± 16.4 (57–115)	91.6 ± 28 (54–157)	0.235	78.1 ± 11.8 (60–93)	77.4 ± 9.5 (63–96)	0.754
Number of ventilations	21.7 ± 2.8 (18–27)	20.2 ± 4 (15–30)	0.18	23.4 ± 6.2 (15–36)	22.2 ± 6.3 (15–39)	0.417	22 ± 6.7 (9–33)	21.2 ± 5.6 (9–30)	0.518
SPO_2_	97.9 ± 1.7 (94–99)	97.5 ± 1 (96–99)	0.368	98 ± 0.8 (97–99)	97.4 ± 1.5 (95–99)	0.23	97.4 ± 1.5 (95–99)	96.9 ± 1.8 (94–99)	0.303
TUG	7.6 ± 1.1 (5.9–9.5)	7 ± 1.2 (5.8–10.4)	**0.019**	6.5 ± 0.8 (5.7–8.4)	6.1 ± 0.8 (5.2–8.2)	**0.016**	7.3 ± 1.1 (5.3–9.1)	7.1 ± 1.2 (6–10.4)	0.657
TUG_imagery	3.9 ± 0.9 (2–5.5)	4 ± 0.9 (2.3–5.8)	0.61	3.9 ± 1.1 (2.7–6)	3.9 ± 1.6 (2.3–7)	0.799	4.2 ± 1.4 (2–6.3)	4 ± 1.6 (2.1–6.5)	0.350
Mental stopwatch	38.4 ± 12 (19.2–59.6)	33.1 ± 12.2 (17.4–55.5)	0.05	31.2 ± 12.9 (4.3–44.7)	29.3 ± 16.7 (−5.2 to 54.5)	0.646	33.7 ± 17 (9.4–67.6)	34.8 ± 20.2 (−0.4 to 65.7)	0.534
KVIQ-20_visual	69.5 ± 12.6 (45–85)	74.2 ± 11 (53–85)	**0.022**	67.2 ± 10.3 (51.00–85.00)	70.4 ± 11.1 (52–85)	0.08	71.7 ± 8.9 (57–85)	77.3 ± 7.1 (63–85)	**0.021**
KVIQ-20_kinesthetic	63.5 ± 20.3 (33–85)	65.2 ± 20.9 (32–85)	0.062	59.9 ± 12 (41–83)	65.1 ± 11.7 (46.00–80.00)	**0.032**	67.1 ± 7.2 (55.00–80.00)	73.3 ± 9.9 (59–84)	0.055
KVIQ-20_total	126.7 ± 31 (79–170)	127 ± 31.5 (90–170)	**0.049**	126.6 ± 19.9 (104–168)	135.5 ± 22 (104–165)	**0.022**	138.9 ± 15 (118–165)	150.7 ± 14.7 (125–169)	**0.009**
MOCA	25.4 ± 2.6 (21–30)	27.5 ± 2.7 (21–30)	**0.007**	25.7 ± 2.9 (21–29)	28.1 ± 1.7 (25–30)	**0.005**	28 ± 2.4 (23–30)	28.4 ± 1.5 (26–30)	0.336

*Note:* Data are reported as median ± standard deviation (minimum–maximum). The increases in KVIQ-20 and MoCA scores and decreases in TUG, TUG imagery, and mental stopwatch minutes are better results. Data in bold are statistically significant.

Abbreviations: ARE, active respiratory exercise group; CGr, control group; DBP, diastolic blood pressure; KVIQ-20, Kinesthetic and Visual Imagery Questionnaire-20; MI-AO, motor imagery+action observation group; MoCA, Montreal Cognitive Assessment; SBP, systolic blood pressure; SpO2, oxygen saturation; TUG, Timed Up and Go test.

## Data Availability

The data presented in this study are available upon request from the corresponding author.
